# Timing of Environmental Enrichment Affects Memory in the House Cricket, *Acheta domesticus*

**DOI:** 10.1371/journal.pone.0152245

**Published:** 2016-04-08

**Authors:** Heather S. Mallory, Aaron F. Howard, Martha R. Weiss

**Affiliations:** 1 Department of Biology, Georgetown University, Washington, D. C., United States of America; 2 Department of Biology, Northeastern Illinois University, Chicago, Illinois, United States of America; University of Guelph, CANADA

## Abstract

Learning appears to be ubiquitous among animals, as it plays a key role in many behaviors including foraging and reproduction. Although there is some genetic basis for differences in learning ability and memory retention, environment also plays an important role, as it does for any other trait. For example, adult animals maintained in enriched housing conditions learn faster and remember tasks for longer than animals maintained in impoverished conditions. Such plasticity in adult learning ability has often been linked to plasticity in the brain, and studies aimed at understanding the mechanisms, stimuli, and consequences of adult behavioral and brain plasticity are numerous. However, the role of experiences during post-embryonic development in shaping plasticity in adult learning ability and memory retention remain relatively unexplored. Using the house cricket (*Acheta domesticus*) as a model organism, we developed a protocol to allow the odor preference of a large number of crickets to be tested in a short period of time. We then used this new protocol to examine how enrichment or impoverishment at two developmental stages (either the last nymphal instar or young adult) affected adult memory. Our results show that regardless of nymphal rearing conditions, crickets that experienced an enriched rearing condition as young adults performed better on a memory task than individuals that experienced an impoverished condition. Older adult crickets (more than 1 week post adult molt) did not demonstrate differences in memory of the odor task, regardless of rearing condition as a young adult. Our results suggest that environmentally-induced plasticity in memory may be restricted to the young adult stage.

## Introduction

Animals can learn a variety of cues, such as odor, color, shape, and pattern, across different behavioral and environmental contexts [1,2]. Learning and memory ability are not fixed traits, but can be affected by the experiences of the animal. For example, adult rats kept in enriched living conditions consisting of extra space, toys, hidden food, and other rats, learn to run a maze more quickly than do those raised in isolation [3]. Despite a rich literature examining the effects of enrichment on adult learning ability (reviewed by [4]), studies examining the extent to which plasticity of learning ability in the adult is affected by experiences in the juvenile stage are relatively few. Enrichment or deprivation may have a greater effect on adult learning and memory if it occurs while the animal is still undergoing post-embryonic development. Studies in mice and cuttlefish suggest that early experiences may not impact adult learning ability, as impoverishment of the early rearing environment did not dampen the response to later environmental enrichment in the adult [5,6]. However, it is known that sensory and motor experiences, as well as stress and interactions with conspecifics that occur from birth well into adulthood, can have important input on the development of the prefrontal cortex in mammals [7]. It is also possible that duration of enrichment may be even more critical than the life stage at which enrichment occurs, as other studies have shown that the duration of early enrichment determines how long its beneficial effects will persist after animals are switched to an isolated condition [8,9]. These somewhat contradictory results leave open the question of whether rearing condition prior to the adult stage can affect plasticity of learning ability and memory retention in the adult. Additionally, the above studies manipulate the rearing environments to extremes, in an understandable effort to reveal differences; isolated individuals are often maintained in 24 hours of dark, with every effort taken to minimize auditory and olfactory stimulations. Although these studies have provided valuable clues about the influence of sensory information on learning and memory, they do not represent realistic differences in sensory experiences that an individual may experience.

Insects are useful model organisms, as they share fundamental behaviors and neurobiological mechanisms with vertebrates, but are less expensive, easier to rear in large numbers, and have smaller, relatively simpler, and more tractable nervous systems [10]. Studies of insect learning and memory have led not only to insights about insect behaviors, but have revealed fundamental principles about the processes underlying these behaviors. For example, studies in the fruit fly *Drosophila* have added to basic knowledge about the genes, neural pathways, and behaviors involved in learning and memory, and as a consequence *Drosophila* is currently recognized as a model organism for many disciplines of biology (reviewed by [11]). However, *Drosophila* are holometabolous insects, which means that they undergo dramatic structural and behavioral changes between the juvenile and adult stages; an egg hatches into a worm-like larva, followed by an outwardly quiescent pupal stage, from which emerges a sexually mature, flying adult. This developmental process differs considerably from the continuous, determinate growth characteristic of vertebrate development. In contrast, hemimetabolous insects undergo incomplete metamorphosis, in which the juvenile resembles the adult in structure and behavior, such that experiences of the juvenile are relevant to the adult stage. Therefore, hemimetabolous insects are better models for examining learning and memory across the lifetime of vertebrates than are holometabolous insects.

Crickets are hemimetabolous insects, and in the last decade many behavioral and neurobiological studies have highlighted their strengths as a research organism. *Gryllus bimaculatus* (the field cricket) can learn olfactory [12,13,14,15] and visual cues [16]. Developmental studies in *Acheta domesticus*, the common house cricket, reveal that the mushroom bodies—paired structures in the insect brain important for olfactory learning and memory—undergo continuous neurogenesis throughout the life of the animal, including the adult stage, and that adult neurogenesis can be stimulated by sensory input [17, 18,19]. Although the function of adult neurogenesis is unclear, it may play an important role in learning and memory in both invertebrates and vertebrates [20]. We chose *Acheta domesticus* as our model organism, based on its hemimetabolous life cycle, and the fact that it exhibits adult neurogenesis, which may be important for adult brain plasticity in both vertebrates and invertebrates.

We first established a new training protocol that allows for large numbers of crickets to be trained and tested in a single day. We then used this new protocol in two experiments. In order to determine whether timing of enrichment has an effect on memory retention in the adult, we housed crickets in enriched, nutritional control, or impoverished conditions at two developmental stages; either the last nymphal instar (experiment 1) or adults less than 1 week post-adult molt (experiment 2). We then maintained the adults in one of the housing conditions above for an additional five days before testing their memory of an olfactory learning task. Using this design, we asked two questions: 1) does previous enrichment or impoverishment affect memory in the adult? and, if so, 2) does the timing of enrichment or impoverishment matter? More specifically, does enrichment or impoverishment that occurs during the nymphal stage (while the animal is still developing) affect adult memory more than enrichment or impoverishment that occurs during the young adult stage (once development is complete)?

## Materials and Methods

### Establishing a Training Protocol

Four-week old cricket nymphs (*Acheta domesticus)* which molted to adulthood in roughly one week, were ordered from PetStuff LLC, maintained in a 20 gallon glass terrarium, and provided with water and Fluker’s orange cube cricket diet. Lights were kept on a 14hr daylight schedule and temperature was maintained at 24°C. Twenty-four hours prior to training, female adults were randomly selected from the terrarium and placed individually in 8 cm x 8 cm x 10 cm transparent plastic boxes with water provided. Food was withheld to motivate foraging behavior in the testing arena. We used only female crickets to control for sex differences observed in pilot studies.

**Fig 1:**
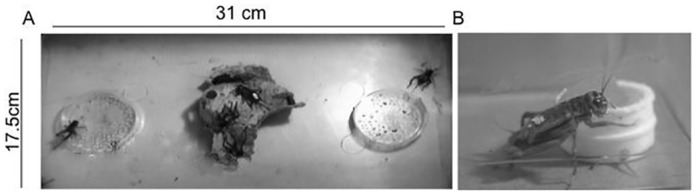
Testing and Training. A) Screen shot of testing arena from video recording B) Training dishes.

To determine whether the crickets preferred either of our test odors, we placed 10 individually marked crickets in a 17.5 cm x 31 cm x 18.5 cm (l x w x h) testing arena. The sides of the arena were wrapped in gray paper to block out interfering visual stimuli, and the arena was lit from above by halogen lamps. Two odor sources were placed in the testing arena, 6 cm from the wall on either end. Odor sources consisted of a 6 cm diameter petri dish containing filter paper soaked in 10μl of essential oil (either rosemary or eucalyptus), and with approximately 100 pin-sized holes in the lid to allow volatiles to disperse. A piece of egg carton in the center of the testing arena provided a resting place for the crickets (Fig 1A). To control for any positional effects, the positions of the odor sources were switched halfway through the 1-hour trial. We video-recorded the trials and later scored them to document number of visits and time spent visiting each odor source. We established an unambiguous criterion to count visits: a visit began when a cricket placed either one of its front legs on the top of the Petri dish, and ended when either one of the front legs of the cricket touched the floor of the testing arena. Commercially available essential oils of two plants, rosemary and eucalyptus, were used as odor sources. We chose these odors because they were plant-based and readily available. As the main goal of this experiment was to establish a new training protocol, rather than investigate the odor preferences of crickets, we felt identity of the odor was less important than our ability to increase an individual’s preference for the odor after pairing it with a reward.

Immediately after odor preference testing was completed, crickets were exposed to a randomly chosen odor, rosemary, in association with Fluker’s orange diet by placing a 2mm plastic lid containing 5μl essential oil of rosemary under another 2mm plastic lid containing the food; the upper lid was perforated with holes to allow the odor to permeate the food without coming into direct contact with it (Fig 1B). Training dishes were kept in the cages with the crickets for 24 hours to give individuals time to associate the presence of food with the odor. Post-training odor preferences were assessed the following day as described above, in the same groups in which they were tested for odor preference prior to training. To test whether crickets were habituating or becoming sensitized to the smell of rosemary, 30 adult female crickets were isolated, tested for odor preference as above, exposed to rosemary odor without food for 24 hours in isolated chambers, and then retested for odor preference in the same groups. Average time spent on each odor source during a trial and number of visits to rosemary and eucalyptus before and after training was compared using paired Student’s T-tests in SPSS.

### Effect of Timing of Enrichment on Adult Memory

All crickets were kept at 24°C on a 14h daylight cycle. Crickets were ordered as four-week old nymphs from PetStuff LLC and maintained communally in a large colony prior to being placed in one of three conditions: Enriched (E), Impoverished (I), or Nutritional Control (NC). 'E' crickets were placed in groups of four in a 8 cm x 8 cm x 10 cm transparent plastic chamber, reared on Fluker’s orange cube diet (which meets the nutritional needs of the crickets) as well as various fruits and grains, and provided with a piece of egg carton for shelter and sticks for climbing. 'I' crickets were placed singly in a 4cm x 4cm x 7cm translucent plastic chamber and reared on a Fluker's orange cube diet only. 'NC' crickets were raised in conditions identical to 'I', but were provided with the same foods as the 'E' crickets to serve as an intermediate control.

We chose an intermediate environment where diet was enriched and social experiences were deprived, as we believed the biggest confound in our impoverished condition was the difference in nutrients. A fourth treatment of socially enriched crickets provided with impoverished diet would have isolated the factor of diet more fully, but would have necessitated increasing the number of treatments from 9 to 16 in order to maintain our crossed experimental design. As we were already near the limits of our capacity to track each cricket individually through the experiment and to generate reasonable sample sizes within a treatment, a compromise was made and the fourth treatment was not included. We believe our NC treatment still serves as an informative intermediate condition, one that is rarely included in studies of environmental effects on learning and memory ability.

**Fig 2:**
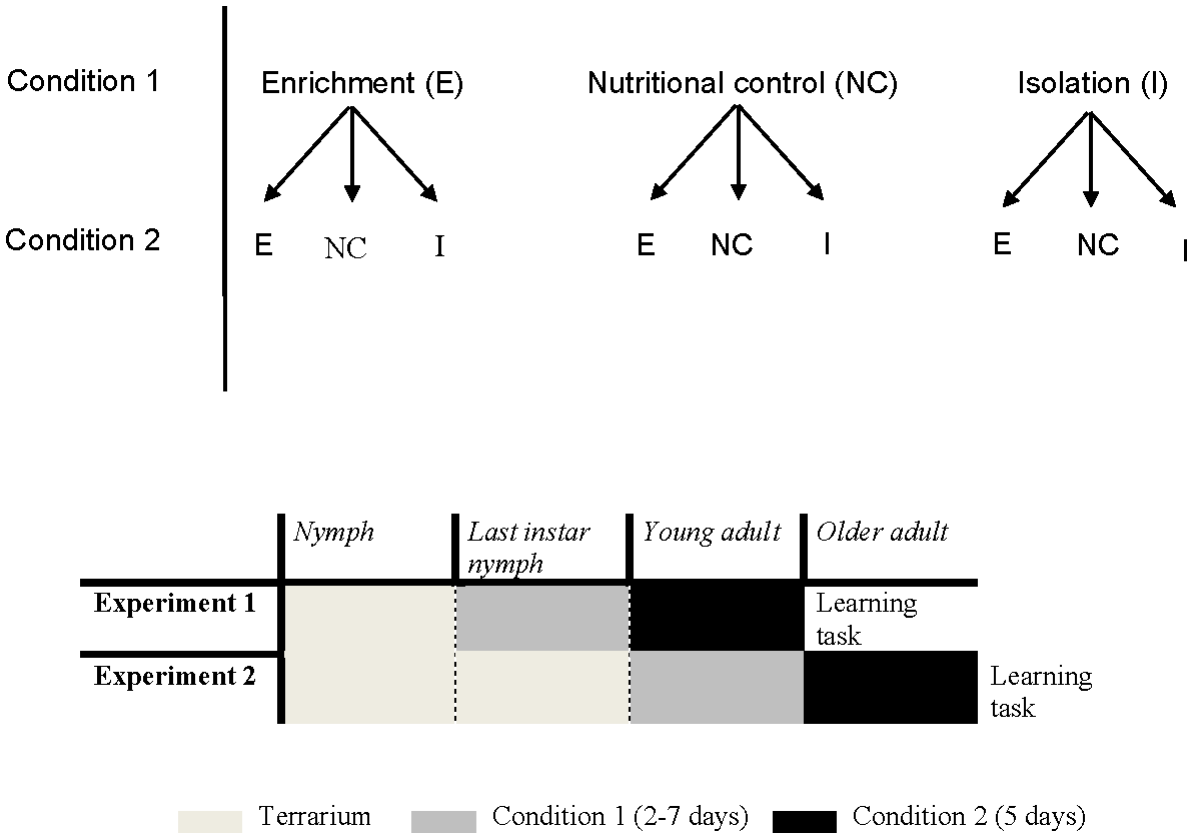
Experimental design. Last instar nymphs (Experiment 1: Nymph Experiment) or young adults (Experiment 2: Adult Experiment) were randomly assigned to one of three conditions: enriched (E), nutritional control (NC), or impoverished (I), (see text for details on conditions). Three to seven days later individuals were randomly assigned to either another or the same condition for five days as an adult.

To determine if rearing conditions had an effect on adult memory, we randomly assigned crickets to one of the above treatments at two different life stages. The full experimental design is shown in Fig 2. For experiment 1, hereafter referred to as the Nymph Experiment, last nymphal instar crickets were maintained in their assigned condition until they molted to the adult stage; time in the assigned condition varied, and ranged from three to seven days (median four days) as we could not predict when nymphs would molt to the adult stage. Newly molted adults were then randomly re-assigned to one of the three conditions and maintained there for five additional days. Thus, a cricket could remain in the same condition or be switched to one of the other two conditions. Crickets assigned to remain in the same treatment were nonetheless transferred to a new cage so that all crickets were handled an equal amount. All cages were kept in the same rearing room throughout the experiment.

**Fig 3:**
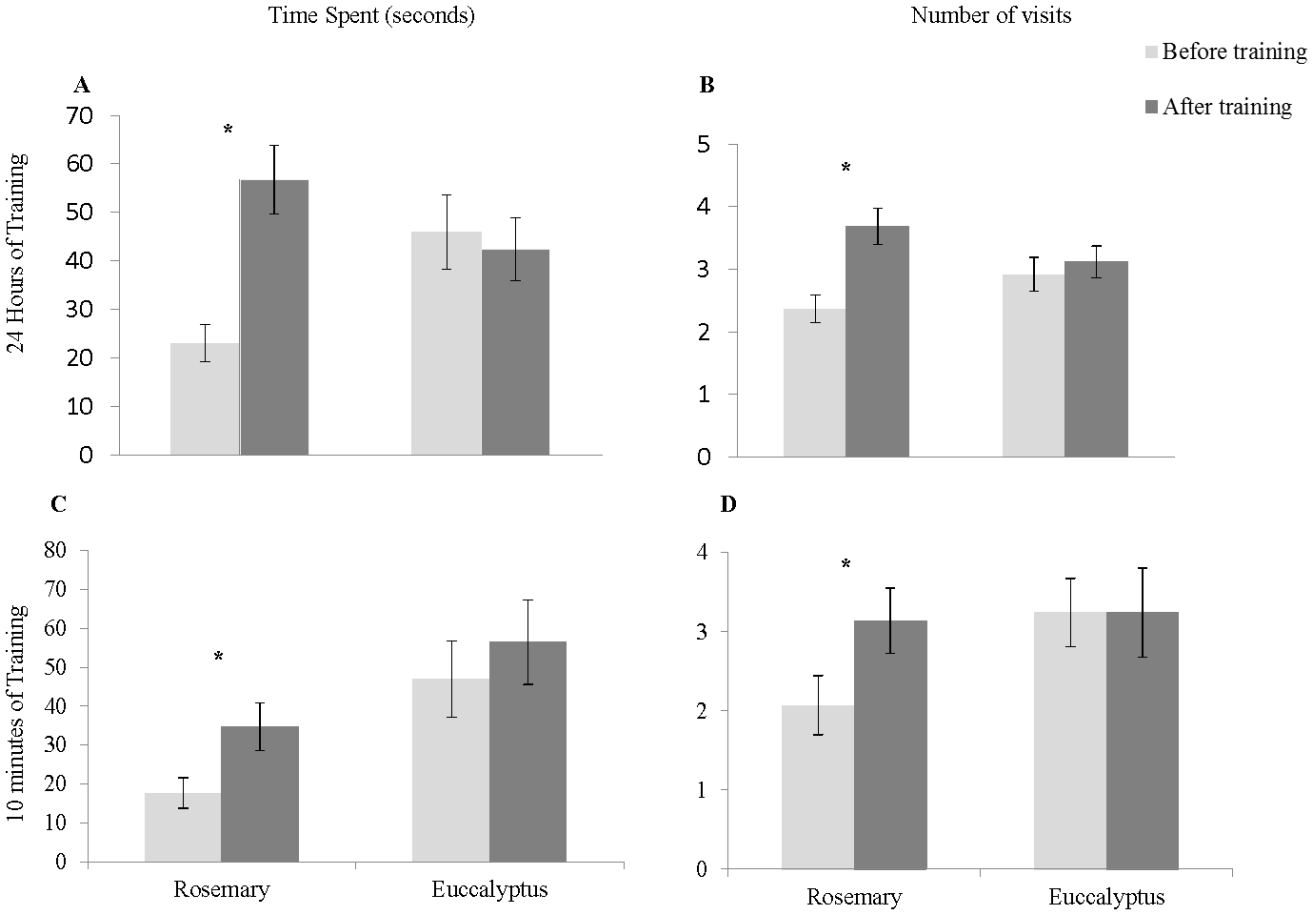
Appetitive conditioning to rosemary. (A, C) Mean ± SE time spent on each odor before and after appetitive conditioning to rosemary. (B, D) Mean ± SE number of visits on each odor before and after appetitive conditioning to rosemary. (A, B) 24 hours of training, (C, D) 10 minutes of training. See Table 2 for details of statistical analysis.

Using the ‘training protocol’ described above, all crickets were given an olfactory memory task on the fifth day after being switched to the second rearing condition. Odor preference testing was followed immediately by ten minutes of training, and post-training preference testing occurred the next day. Crickets were food-deprived between training and testing. Non-trained and trained groups were kept separated during odor preference training, but treatments were mixed within the group of ten crickets. Crickets were marked as in the experiment above to track individuals, and the observer measuring visits was blind to treatment during scoring. Training time was reduced to ten minutes after pilot tests using adult females demonstrated that a reduction in training time still resulted in a trained preference for rosemary (Fig 3). Twenty-four hours prior to odor preference testing, food, but not water, was withheld to motivate search.

Experiment 2, hereafter referred to as the Adult Experiment, was designed to determine whether housing conditions at the young adult stage, just after the adult molt when development was presumably complete, would affect memory in older adults. Comparisons between this experiment (Adult Experiment) and the previous experiment (Nymph Experiment) can reveal if the timing of these experiences is important. Young adult crickets (less than three days since adult molt) were assigned to one of the three conditions for three to seven days (in order to replicate the variation in duration of exposure in the Nymph Experiment), after which they were randomly assigned to one of the three conditions for five additional days. All crickets were then given an olfactory memory task in exactly the same manner as in the Nymph Experiment.

To take full advantage of our crossed experimental design, we compared memory across treatments with a single metric. Therefore, instead of comparing number of visits or time spent visiting each odor source independently within each treatment, which would test only whether memory was retained within each treatment, we used the change in time spent on rosemary (total time on rosemary after training minus total time spent on rosemary before training) as the response variable. This ‘preference index’ was calculated for each individual cricket, and has been used in several cricket studies as a metric of learning ability and memory [12,13,14]. Data were non-normal with heteroscedasticity, and were therefore square root-transformed across all treatments prior to ANOVA. After transformation, the datasets met the assumptions of normality (KS tests: Nymph experiment D = 0.11, p = 0.12; Adult experiment D = 0.12, p = 0.28) and homoscedasticity (Levene’s tests: Nymph experiment F_(8,193)_ = 1.5, p = 0.14; Adult experiment F_(8,77)_ = 1.05, p = 0.40), and ANOVAs were carried out using first condition and second condition as the main effects and time spent in first condition as a covariate, followed by Tukey’s Post-hocs if overall differences were detected. Cohen’s ƒ^2^ was calculated to measure the effect size of each factor [21]. Statistics were run in R: A Language and Environment for Statistical Computing (R Core Team).

## Results

### Establishing a Training Protocol

Crickets learned to associate the odor of rosemary with a reward when provided with 24 hours of training (Fig 3A and 3B, Table 1). Both the average time spent on rosemary (paired T-test t_50_ = -4.86, p = 0.0001) and the number of visits to rosemary (paired T-test t_50_ = -4.0, p = 0.0002) increased after training. The number of visits and time spent on the non-trained odor, eucalyptus, did not change. Similar results were obtained using 10 minutes of training (Fig 3C and 3D). The time spent on rosemary (paired T-test t_28_ = -4-2.49, p = 0.019) increased after training, and the number of visits to rosemary increased, although this result was statistically borderline (paired T-test t_28_ = -2.02, p = 0.05). The number of visits and time spent on the non-trained odor, eucalyptus, did not change. Crickets that were maintained in their cages for 24 hours with the smell of rosemary but no food reward did not show a change in time spent on rosemary (paired T-test t_20_ = 0.318, p = 0.754), number of visits to rosemary (paired T-test t_20_ = 1.61, p = 0.123), time spent on eucalyptus (paired T-test t_20_ = -0.277, p = 0.785) or number of visits to eucalyptus (paired T-test t_20_ = 0.318, p = 0.754) in preference testing.

**Table 1:** Paired-t tests showing change in time spent at and number of visits to the odor sources with 24 hours of training and 10 minutes of training.

10 minutes training			
	df	t	p-value
Time on Rosemary	28	-2.489	0.019
Time on Eucalyptus	28	-0.639	0.528
Number of visits to Rosemary	28	-2.018	0.053
Number of visits to Eucalyptus	28	0	1.00
**24 hours training**			
Time on Rosemary	50	-4.857	0.0001
Time on Eucalyptus	50	0.369	0.714
Number of visits to Rosemary	50	-4.001	0.001
Number of visits to Eucalyptus	50	-0.508	0.614

### Effect of Timing of Enrichment on Adult Memory

#### Nymph experiment

**Fig 4:**
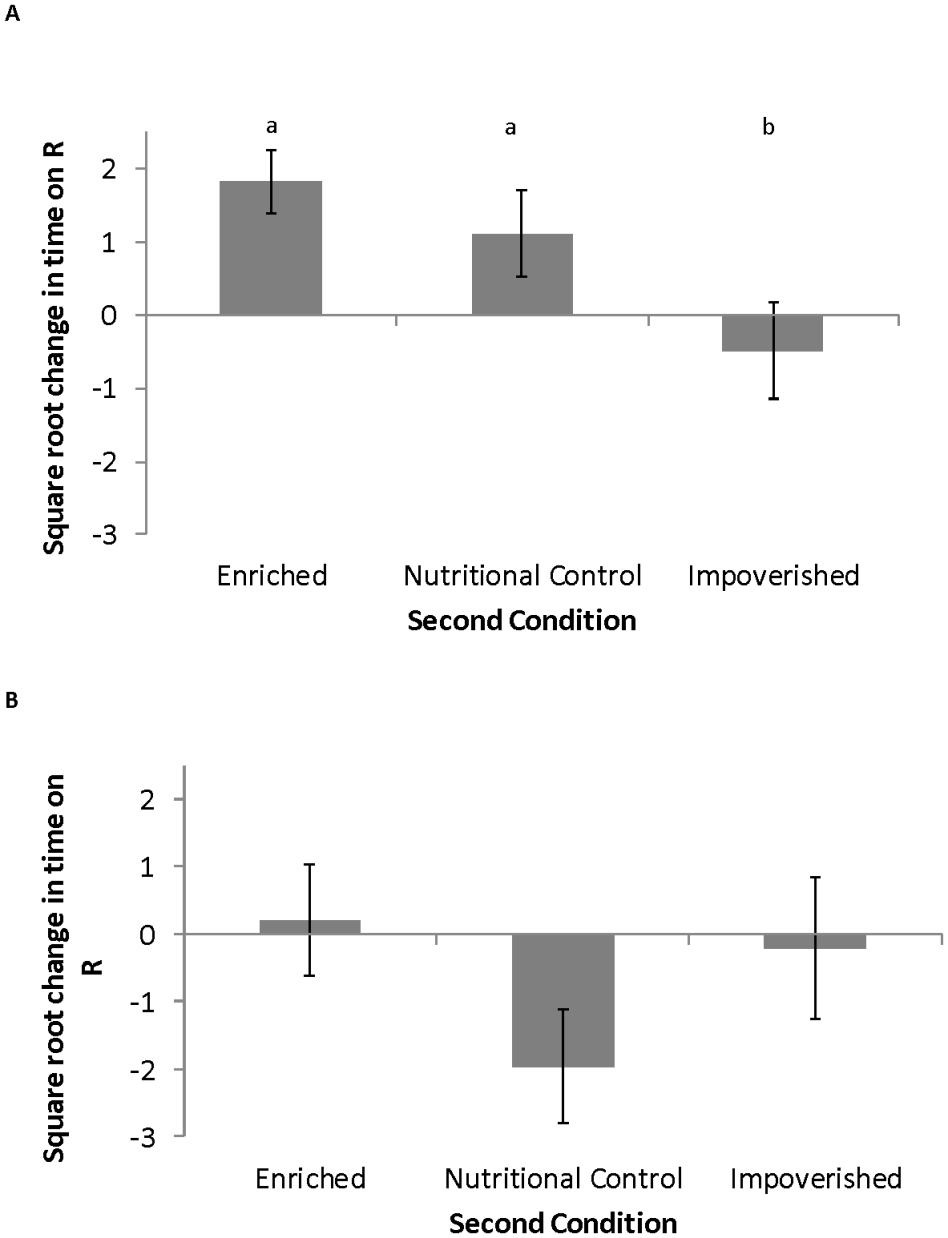
Enrichment effects on adult memory. A) Nymph Experiment: Last instar nymphs were placed in one of the three conditions (E, NC, I) and then on the day an individual molted to the adult stage it was randomly placed in one of the three conditions for 5 additional days. An effect of second condition was detected on change in time spent on the training odor, rosemary (ANOVA F_(2,8)_ = 4.776, p = 0.009. Sample size for each group (Enriched, Nutritional control, and Isolated) is 38, 40, and 57 crickets respectively. Letters indicate significant differences at p = 0.01 level detected the Tukey’s Post-hoc. B) Adult Experiment: Young adults maintained in one of the three conditions (E, NC, I) for three to seven days were then randomly placed in one of the three conditions for five additional days. No significant effect of second condition was detected. Sample size for each group (Enriched, Nutritional control, and Isolated) is 40, 21 and 25 crickets respectively.

Rearing condition at the last nymphal instar had no effect on memory in the young adults (Table 2, F_(2,8)_ = 1.40, p = 0.869). Regardless of whether they had been isolated or enriched as nymphs, crickets that were enriched as young adults increased their preference for rosemary more than crickets isolated as young adults (Table 2 and Fig 4, F_(2,8)_ = 4.776, p = 0.009, Tukey's post-hoc, E vs I, p = 0.0306). Therefore, isolation at the last nymphal instar did not reduce the ability of a young adult to respond to the enriched condition as an adult. Similarly, enrichment at the last nymphal instar did not carry over to the young adult stage, leading to a young adult from the impoverished condition performing as well on the memory task as a young adult in the enriched condition. Young adults in the nutritional control condition did not significantly differ from enriched young adults, but increased their preference for rosemary more than crickets isolated as young adults (Tukey's post-hoc, NC vs I, p = 0.0315) regardless of their housing condition as last instar nymphs.

**Table 2:** Summary of statistical analysis run in SPSS. Significant effects are at the p<0.05 level are bolded. See text for detailed descriptions of conditions.

Experiment	Factor	df	F	p-value	power	Cohen’s ƒ^2^
*Experiment 1*: *Nymphs*	Condition 1 (nymph)	2	0.140	0.869	0.148	0.00026
	Condition 2 (young adult)	2	4.776	**0.009**	**0.562**	**0.02711**
	Condition 1 * Condition 2	4	1.356	0.251	0.253	0.01182
	Days in condition 1	1	0.343	0.559	0.019	0.00038
*Experiment 2*: *Adults*	Condition 1 (young adult)	2	0.110	0.896	0.025	0.00176
	Condition 2 (older adult)	2	1.256	0.290	0.022	0.00272
	Condition 1 * Condition 2	4	1.861	0.126	0.019	0.00049
	Days in condition 1	1	6.057	**0.016**	**0.562**	**0.0560**

No interactions between first (nymph) and second (young adult) condition were detected (Table 2, F_(2,8)_ = 1.356, p = 0.251). The amount of time spent in first condition as a last instar nymph, which was run as a covariate, did not have a significant effect (Table 2, F_(2,8)_ = 0.343, p = 0.559).

#### Adult experiment

**Fig 5:**
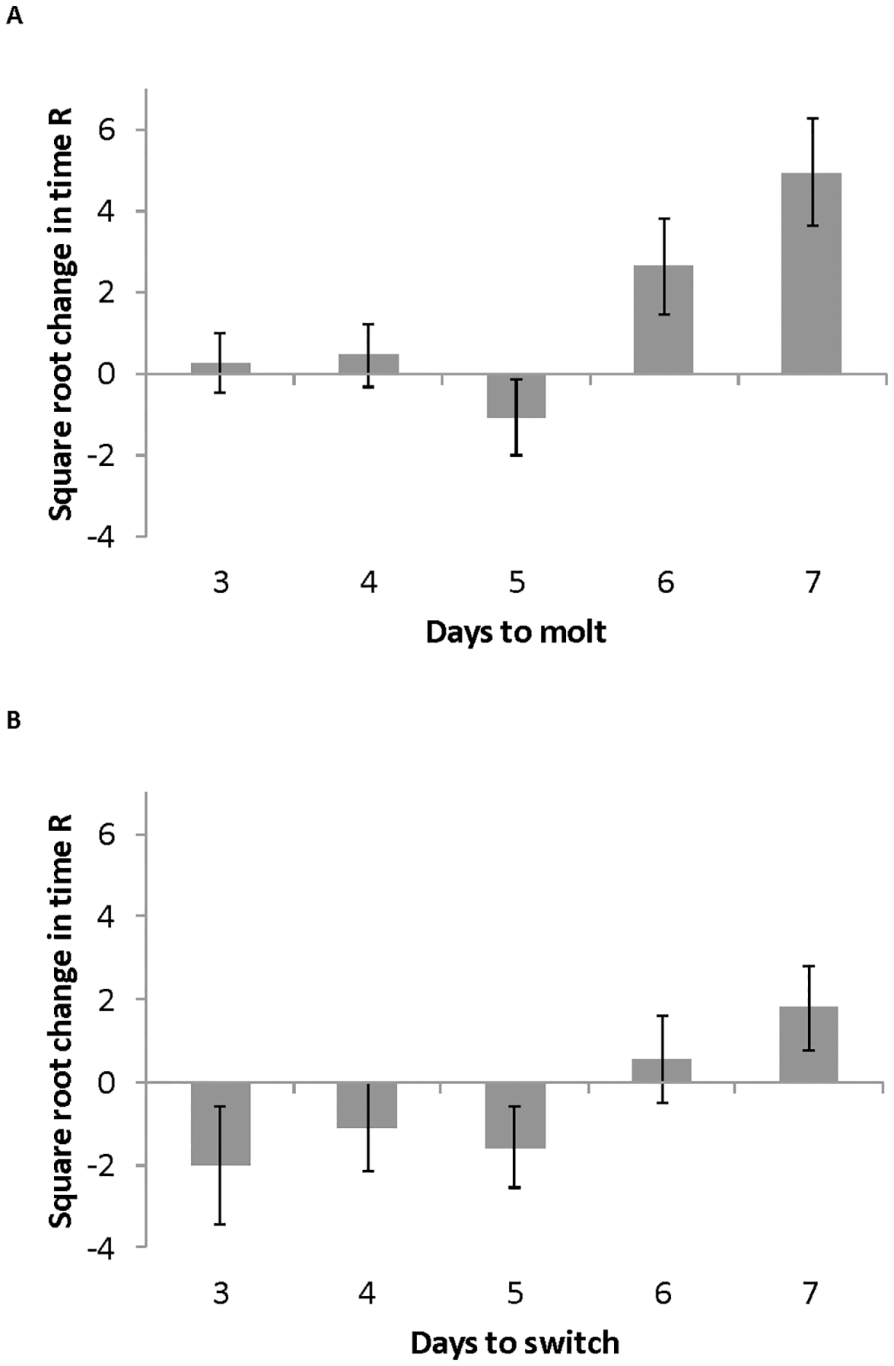
Time spent in first condition. A) Last instar nymphs were placed in one of the three conditions (E, NC, I) and then on the day an individual molted to the adult stage it was randomly placed in one of the three conditions for five additional days. Days in first condition were not correlated to change in time spent on rosemary. B) Young adults were in one of the three conditions (E, NC, I) for three to seven days and then randomly placed in one of the three conditions for five additional days. Duration of time spent in first condition, a co-variate in the ANOVA, was significant (F_(1,9)_ = 6.057 p = 0.016).

Neither experience at the young adult (condition 1) or older adult stage (condition 2) lead to relative differences in memory (Table 2, Fig 4, F_(2,8)_ = 0.110, p = 0.896 and F_(2,8)_ = 1.861, p = 0.126 respectively). No interactions between first (nymph) and second (young adult) condition were detected (Table 1, F_(2,8)_ = 1.861 p = 0.126). The co-variate in the ANOVA, days spent in first condition, was significant (Table 1 F_(1,9)_ = 6.057 p = 0.016). Regardless of housing condition (E, NC or I) in the young adult stage, change in time spent on rosemary also increased as time spent in the first condition increased (Fig 5).

## Discussion

### Establishing a new training protocol

We were able to train crickets to associate the odor of rosemary with a food reward, as both time spent and number of visits to rosemary increased significantly after training (Fig 3). Time spent on the non-trained odor, eucalyptus, did not change between testing and training, indicating that crickets increased their foraging effort on the trained odor, but did not abandon the non-trained odor. An alternative explanation for the observed increase in number of visits to and time spent on rosemary could be that crickets are visiting the odor that is more familiar, rather than truly associating the odor of rosemary with a food resource. However, crickets that were exposed to the odor without a food resource neither increased nor decreased their time spent on either odor during preference testing, showing that neither habituation nor sensitization explains the increased time spent on rosemary following training with food reward. Reciprocal training was not performed, as it has already been well established that crickets are capable of olfactory learning [12,13,14,15], and the goal of this experiment was to establish a new protocol, not to test the olfactory learning capabilities of crickets. This newly developed protocol could be especially useful when large-scale experiments with multiple treatments will be used.

Testing the odor preference of the crickets as a group could result in a lack of independence of data points; the presence of a cricket at the odor source may either recruit others to the dish, or deter other crickets from visiting the odor source [22,23]. However, we do not believe that either recruitment or deterrence was a contributing factor in our study. Firstly, there was rarely more than one cricket at an odor source at any one time, suggesting that recruitment is unlikely. In a survey of five randomly selected trials, which included 79 visits to an odor source, we observed just seven instances of two crickets on the odor source at the same time, and in only one of those instances did the crickets directly interact. Furthermore, when there were two crickets on an odor source at once, the average length of the visit was not different than when a single cricket was present. The fact that crickets rarely shared an odor source may suggest deterrence; however, during the course of a trial, visits to an odor source comprised a small fraction of the overall trial time (usually less than 5% of the total time) as visits tended to be short in duration. Thus the unoccupied odor sources were available for visits the vast majority of the time of the trial. Although this does not rule out opportunities for social learning during the one-hour testing period [23], opportunities for learning during the group testing would be similar across treatments, and therefore unlikely to be a factor in our final results. Additionally, in both the Nymph and Adult experiments, crickets from multiple treatment groups were represented in any given odor preference test; therefore, any influence that an individual cricket may have had on the behavior of another would be randomized across treatment groups. Finally, replicated goodness-of-fit tests indicate that the number of visits to either odor is not influenced by grouping (Nymph experiment: $\chi_{84}^{2}$ = 4.77, P = 1; Adult experiment: $\chi_{24}^{2}$ = 8.76, P = 0.99). Although we cannot fully rule out issues of non-independence, we feel that the benefits of this design, which include generating a larger sample size than possible with single testing and increasing overall activity of the crickets (crickets alone in the arena rarely moved), outweigh the possible influence of interactions between crickets on the odor source.

### Effect of Timing of Enrichment on Adult Memory

To our knowledge, no other study has used a fully crossed experimental design—with permutations across enriched, impoverished, and nutritional control environments—in concert with relatively moderate environmental manipulations, to examine the effect of previous environmental condition on plasticity of learning ability and memory in adult insects. Our results suggest that the answers to our originally posed questions: 1) does previous enrichment or impoverishment affect memory in the adult? and 2) does enrichment or impoverishment that occurs during the nymphal stage (while the animal is still developing) affect adult memory more than enrichment or impoverishment that occurs during the young adult stage (once development is complete)? are both no. It is more likely that environmentally-induced plasticity in adult memory may be correlated with the age of the cricket.

Although our overall statistical power was low, we are confident that our results are informative. They show that the time spent and effect of rearing condition as young adults has a small but statistically significant effect on memory, and that no other effects are detected, or would be detected, even given a significantly higher sample size. A power analysis shows that for the Nymph Experiment, four times the sample size is required to reach a power of 0.8 for the main effects in the ANOVA, and that even at 20 times the sample size for the adult experiment, power is still low (0.014, 0.312, 0.066 for condition 1, condition 2, and interaction, respectively). Even three times our sample size would have be hard to achieve, let alone 20 times our sample size, because for a cricket to be included in the analyses it first had to survive two weeks in the different conditions, and then visit both odor sources both before and after training; to achieve current sample sizes required running over 2700 crickets through the experiment. These numbers underscore the difficulty of obtaining large sample sizes for these types of experiments, and demonstrate that single testing of crickets would have been functionally impossible. Future experiments to develop and refine high-throughput behavioral testing are necessary for the advancement of these questions.

Our finding that rearing condition at the last nymphal instar did not affect memory retention in young adults is in line with the results of several other studies. Work on mice and cuttlefish suggest that the most recent condition is more important than previously experienced conditions, with individuals coming from an enriched environment performing better at behavioral tasks, regardless of earlier impoverishment [5,6]. Our results from the Adult Experiment, which indicate that duration in young adult condition may have an effect on behavioral plasticity, are also in line with published reports. Studies in rats examining the effect of enrichment of different durations and at different ages have shown that both have an influence, with longer durations of enrichment leading to greater behavioral differences, and older rats showing reduced effects of enrichment [8,24]. In mice, a longer duration of enrichment results in greater behavioral differences that persisted for longer after switching back to standard rearing conditions [9]. Perhaps if our crickets had been maintained in their first-condition treatments for longer, we would have seen carryover effects in the adults. It is worth noting that the crickets in our study spent most of their nymphal stage in breeder conditions, which are quite different from any in our study, and could be considered enriched given that breeders keep crickets in large number in large tanks. As these early conditions were the same for all crickets in our study, this fact does not change our results or interpretations, but leaves open future research questions. If crickets are bred in more enriched vs less enriched environments, would the results of our study differ? Future studies directly manipulating the amount of time in each condition are necessary to tease apart how timing and duration of enrichment affect plasticity of learning ability and memory in adults.

Which element of the enriched or impoverished condition explains the apparent differences in memory? Our rearing conditions were not as drastically different from each other as those in other studies manipulating sensory experiences. In other studies, impoverished conditions include 24 hours of complete darkness or only dim light, with minimal noise, smells, and other sensory stimulation [8,25]. This almost complete lack of sensory stimulation differs from our impoverished condition, where animals were exposed to light through the translucent cage, and could likely smell and hear other crickets nearby. We created environments that differed less dramatically in order to examine the effects of relative differences in sensory information, in situations that are more similar to what animals may experience in nature, rather than presence or virtual absence of information. Our results highlight how sensitive animals can be to differences in sensory information.

Although our enriched and impoverished conditions were not as dramatically different as those used in other studies, there were still many differences between the two treatments, including cage size, number of individuals per cage, presence or absence of objects for hiding under or climbing on, complexity of visual environment, and different diets which provided not only different nutrients but also novel smells and textures. As crickets subjected to the nutritional control condition did not differ from the enriched condition, but did differ from the isolated condition, diet effects may explain a large portion of the variation we saw in memory. Most studies examining the effect of rearing environment on learning include only an enriched and an impoverished condition, or rarely include standard laboratory conditions as a comparison group. Without an intermediate condition it is difficult to determine if enrichment correlates with an increase in learning ability and memory, if impoverishment correlates with a decrease, or if the two act together. Our inclusion of an intermediate treatment allowed us to determine that diet alone can explain the much of the differences observed between the enriched and impoverished conditions, a key element that is missing in many previous studies. However, it is likely that, in addition to diet, interactions with other individuals, and structural differences between the housing conditions may have additive effects on plasticity of learning ability and memory in the adult.

Understanding what elements of the enriched condition contribute to the observed differences seen in memory can help us to understand what cues are of primary influence for adult behavioral plasticity. Studies of *Drosophila* have shown that living space available per animal is a primary factor in explaining differences in mating behavior between enriched and isolated flies [26]. In our study the crickets housed in all conditions had the same ‘floor space’ (per square cm area); enriched cages had a floor area of 16cm^2^ shared by 4 crickets; impoverished and nutritional control crickets were housed individually in cages of 4cm^2^ floor area. However, the enriched cages were taller and contained sticks and an egg carton for climbing, allowing enriched crickets to move vertically in the space and potentially obtain more exercise. Opportunity to exercise has been shown to be an important component of the enriched environment [27], and could be in our study as well. Social interactions may also be a key part of the enriching environment, although a study in rats did not find support for the role of social interactions such as play in the effects of enrichment [28]. Exposure to different tastes and smells, as through the varied diet, can also affect neural systems and behavior [29,30]. Clearly, many different factors act together to bring about the effects of the enrichment [4], and future studies are needed to pick apart these different elements.

Our results have important implications not only for understanding plasticity of memory in adult animals, but also for how animals are maintained in laboratories. Laboratory and captive rearing environments are depauperate relative to the natural environment, and this disparity has fundamental implications for researchers. Enrichment in the laboratory setting has been used, to our knowledge, only as an experimental variable, and has yet to be adopted as common practice for rearing of research animals, particularly invertebrates. Given the sensitivity of animals to their rearing conditions, careful consideration and documentation of rearing condition is necessary, particularly when studying the cognitive abilities of animals.
